# Socioeconomic deprivation and genetic ancestry interact to modify type 2 diabetes ethnic disparities in the United Kingdom

**DOI:** 10.1016/j.eclinm.2021.100960

**Published:** 2021-06-14

**Authors:** Shashwat Deepali Nagar, Anna María Nápoles, I. King Jordan, Leonardo Mariño-Ramírez

**Affiliations:** a School of Biological Sciences, Georgia Institute of Technology, Atlanta, GA, USA; b National Institute on Minority Health and Health Disparities, 3 Center Drive, Building 3, Floor 5, Bethesda, MD 20814, USA; c PanAmerican Bioinformatics Institute, Cali, Colombia; d IHRC-Georgia Tech Applied Bioinformatics Laboratory, Atlanta, GA, USA

## Abstract

**Background:**

Type 2 diabetes (T2D) is a complex common disease that disproportionately impacts minority ethnic groups in the United Kingdom (UK). Socioeconomic deprivation (SED) is widely considered as a potential explanation for T2D ethnic disparities in the UK, whereas the effect of genetic ancestry (GA) on such disparities has yet to be studied.

**Methods:**

We leveraged data from the UK Biobank prospective cohort study, with participants enrolled between 2006 and 2010, to model the relationship between SED (Townsend index), GA (clustering principal components of whole genome genotype data), and T2D status (ICD-10 codes) across the three largest ethnic groups in the UK – Asian, Black, and White – using multivariable logistic regression.

**Findings:**

The Asian group shows the highest T2D prevalence (17·9%), followed by the Black (11·7%) and White (5·5%) ethnic groups. We find that both SED (OR: 1·11, 95% CI: 1·10–1·11) and non-European GA (OR South Asian versus European: 4·37, 95% CI: 4·10–4·66; OR African versus European: 2·52, 95% CI: 2·23–2·85) are significantly associated with the observed T2D disparities. GA and SED show significant interaction effects on T2D, with SED being a relatively greater risk factor for T2D for individuals with South Asian and African ancestry, compared to those with European ancestry.

**Interpretation:**

The significant interactions between SED and GA underscore how the effects of environmental risk factors can differ among ancestry groups, suggesting the need for grou*p-*specific interventions.

**Funding:**

This work was supported by the National Institutes of Health (NIH) Distinguished Scholars Program (DSP) to LMR and the Division of Intramural Research (DIR) of the National Institute on Minority Health and Health Disparities (NIMHD) at NIH.

**Research in context**

**Evidence before this study**

Type 2 diabetes (T2D) is known to disparately impact minority ethnic groups in the United Kingdom (UK). Diabetes UK reports that the prevalence of T2D is two to four times as high in Asian and Black ethnic groups compared to the White ethnic group. Socioeconomic deprivation (SED) is considered to contribute to T2D ethnic disparities in the UK. Higher deprivation has been associated with higher T2D prevalence in Asian and Black ethnic groups. A role for genetic ancestry (GA) in T2D ethnic disparities is more contentious. Studies in the US and Latin America have suggested that African and Native American ancestry are risk factors for T2D among Black and Hispanic populations, but these effects are often attenuated when SED is controlled for. To our knowledge, there have been no studies on the effect of genetic ancestry (GA) on T2D health disparities in the UK.

**Added value of this study**

Since ethnic groups are socially defined based on shared heritage and culture, they are thought to be imprecise proxies for biological factors that impact health outcomes, including genetic diversity. Genetic ancestry (GA) better captures the genetic diversity among population groups. Accordingly, a focus on GA can be used to disentangle the socioenvironmental and genetic dimensions of T2D health disparities. A focus on interactions between GA and socioenvironmental factors has been prioritized as a means to elucidate grou*p-*specific risk factors and thereby promote health equity.

**Implications of all the available evidence**

Results of this study suggest that both SED and GA are important risk factors for T2D diabetes, consistent with a multifactorial genetic and environmental etiology. Non-European GA and SED interact to effect the risk of T2D. SED is a relatively greater risk factor for T2D for individuals with South Asian and African ancestry, compared to those with European ancestry. The observed interaction suggests the need for targeted interventions that recognize the distinct implications of SED for T2D risk across ethnic groups in the UK.

Alt-text: Unlabelled box

## 1 Introduction

Diabetes is rapidly becoming a global pandemic, largely due to increasing rates of obesity [1]. It is estimated that by 2030, diabetes will impact ~5·5 million individuals in the United Kingdom (UK), with type 2 diabetes (T2D) accounting for ~90% of all cases [2]. T2D is a health disparity that disproportionately impacts minority ethnic groups [3]. Asian and Black ethnic groups in the UK have approximately two to four times the T2D prevalence compared to White and other ethnic groups [2]. Efforts to mitigate health disparities of this kind are both a social imperative and a pressing scientific challenge.

It should be noted that studies of health disparities in the UK often rely on the ethnicity categories used by the National Health Service (NHS) [4]. NHS ethnic categories include six ethnic groups – Asian, Black, Chinese, Mixed, White, and Other – and a distinct ethnic background within each group. UK ethnic group classifications make no distinction between the related concepts of race and ethnicity [5]. Accordingly, the ethnic group labels used in the UK may correspond to racial group labels used in other countries, such as the United States.

T2D is a complex common disease caused by a multifactorial interplay between social, environmental, and genetic factors, all of which contribute to T2D health disparities [6,7]. Accordingly, efforts to elucidate the risk factors associated with T2D ethnic disparities require an integrated approach that considers social, environmental, and genetic components together. An integrated approach of this kind is further distinguished by its potential to characterize how interactions between genetic and environmental factors contribute to disparate health outcomes. Indeed, gene-by-environment interactions have been prioritized for health disparities research [8,9].

Socioeconomic deprivation (SED) is widely considered an important risk factor for T2D ethnic health disparities [[10], [11], [12]]. Lifestyle conditions associated with higher SED – psychosocial stress, restricted autonomy, and limited access to healthy food, exercise facilities, and health services – have been shown to modify risk for T2D [[13], [14], [15]]. In the UK, SED has been associated with a greater T2D prevalence among minority Asian and Black populations than among those identifying as White [16,17]. Genetic differences between ethnic groups, owing to their different ancestral origins, have also been associated with T2D disparities [11] .In the US and Latin America, both African and Native American genetic ancestry (GA) have been associated with T2D disparities in Black and Hispanic populations [[18], [19], [20], [21], [22]]. However, the inclusion of SED has been shown to attenuate the effect of GA on T2D status in these populations [11,20,22]. To our knowledge, there have been no studies that simultaneously consider the impact of GA and SED on T2D ethnic disparities in the UK.

GA provides a number of advantages for health disparities research. Ethnic groups are socially constructed and co-vary with both socioenvironmental and genetic factors. GA inference can be used to stratify populations based on evolutionary genetic diversity alone. A focus on GA can thereby allow for the disambiguation of the genetic and socioenvironmental dimensions of ethnic health disparities. Joint consideration of GA, SED, and their interactions can be used to tailor population-level interventions aimed at mitigating health disparities [8,9].

The objective of this study was to investigate the joint effects of SED and GA on T2D ethnic disparities in the UK. Leveraging the UK Biobank, a large prospective cohort study with genetic and environmental data from more than 500,000 participants, we modeled the relationship between SED, GA and T2D across the three largest ethnic groups in the UK – Asian, Black, and White – using multivariable logistic regression [23]. GA groups were delineated by clustering genetic principal components analysis data, yielding discrete and coherent groups that capture the genetic diversity of the study cohort, thereby isolating genetic from socioenvironmental effects on T2D.

## 2 Methods

### 2.1 Study cohort

The cohort for this study was obtained from the UK Biobank, a prospective cohort study set up to investigate the lifestyle, environmental, and genetic determinants of a range of important diseases of adulthood for participants aged between 40 and 70 years collected between 2006 and 2010 [23]. The UK Biobank database contains phenotypic and genotypic information on more than 500,000 participants over multiple waves of collection. Participants provided information in the form of completed questionnaires, nurse-led interviews, medical assessments, and biological samples. Participant DNA was extracted from 850 µL buffy coat aliquots, derived from 10 ml of whole blood, and participant whole genome genotypes were characterized using the UK Biobank Axiom Array or UK BiLEVE Axiom Array as previously described [24]. The study adheres to RECORD reporting guidelines.

### 2.2 Population attributes and data filtering

We extracted the following information for UK Biobank participants: (1) age (Field 21,003: Age when attended assessment center) [25], (2) sex (Field 31: Sex) [26], (3) Townsend deprivation index (Field 189: Townsend deprivation index at recruitment) [27], (4) ethnic group and background (Field 21,000: Ethnic background) [28], (5) ICD-10 codes (Fields 41,270: Diagnoses – ICD10) [29], and (6) genetic principal components (Field 22,009: Genetic principal components) [30]. As not all of these data fields were available for all participants, the final analysis cohort was constructed by merging these datasets (Supplementary Figure 1). SDN was responsible for accessing, analysing, and curating the datasets involved in the study.

UK Biobank participants self-identified as belonging to one of six ethnic groups (Asian, Black, Chinese, Mixed, White, or Other), and a distinct ethnic background within each group, at the time of enrollment. We consider the three largest ethnic groups for analysis: Asian, Black, and White. The corresponding ethnic backgrounds for the ethnic groups considered for our analyses were: Asian (Indian, Pakistani, Bangladeshi, Any other Asian background), Black (Caribbean, African, Any other Black background), and White (British, Irish, Any other white background).

To study levels of SED, we use the Townsend index of deprivation, a widely used measure of SED that is known to be associated with worse health outcomes [31]. The Townsend index is a composite metric that incorporates (1) unemployment, (2) non-car ownership, (3) non-home ownership, and (4) household overcrowding in a given area [32]. Higher (positive) values of the index indicate high material deprivation, whereas lower (negative) values indicate relative affluence. The cutoff values for the SED quintiles were −3·95, −2·80, −1·37, and 1·23, while those for the SED terciles were −3·17 and −0·68.

### 2.3 Type 2 diabetes prevalence

UK Biobank participants’ case or control status for type 2 diabetes (T2D) was determined using ICD-10 diagnosis codes curated following the phecode scheme defined by the PheWAS consortium [33]. The phecode scheme provides disease-specific inclusion and exclusion criteria ICD-10 codes for generating case/control cohorts from electronic health records. This approach allows investigators to define clearly distinct case and control cohorts that can be compared confidently. For example, when studying participants with T2D, participants with type 1 diabetes are removed from the control cohort to avoid any overlapping environmental/genetic signals that might be common to both. This improves power to detect any signals for a condition of interest. The phecode scheme to define case and control cohorts using ICD-10 codes was validated by investigating phenotype reproducibility with the gold standard ICD-9-CM phecode map and by conducting a PheWAS to replicate older, well-known results [33]. Here, inclusion ICD-10 codes were first used to generate the T2D case cohort, and exclusion codes were subsequently used to remove individuals with related conditions from the remaining control cohort. The T2D phecode (250·2) inclusion and exclusion ICD-10 codes can be found at https://phewascatalog.org/phecodes_icd10. Participants T2D case and control status were used to calculate crude T2D prevalence values for ethnic groups and backgrounds as the percent of cases in each group. Crude prevalence values were used owing to the fact that age and sex were included as covariates in all T2D models.

### 2.4 Genetic ancestry inference

UK Biobank participants self-identify as belonging to ethnic groups based on shared culture and heritage. In other words, ethnic groups are socially constructed and thus may not serve as reliable proxies for genetic diversity [34]. Patterns of genetic diversity among UK Biobank participants were characterized by principal components analysis (PCA) of whole genome genotypes as previously described [23]. Genetic ancestry groups were defined by clustering the first three principal component values from the genetic PCA data. Two different clustering approaches were used to generate (1) continuous genetic ancestry groups and (2) coherent genetic ancestry groups. Continuous genetic ancestry groups were characterized using the k-means clustering algorithm, implemented in the function `kmeans` in R v3·6·1 ([35], using *k* = 3. The value of *k* was set to three (*k* = 3) to identify three clusters in the PCA data to match the three self-identified ethnic groups under consideration. The resulting groups included all individuals (and are therefore dubbed ‘continuous genetic ancestry groups’). Coherent genetic ancestry groups were characterized using the density-based clustering algorithm HDBSCAN [36] implemented in the python module ‘hdbscan’. The clustering function was run with a minimum cluster size (`min_cluster_size`) set to 1000 individuals to extract large, coherent clusters from the data. Density-based clustering only categorizes a subset of participants into ancestry clusters, while marking the rest as uncategorized. In excluding participants that are not tightly clustered, we were able to obtain coherent and highly distinct genetic ancestry clusters. The resulting GA groups are distinguished by systematic (correlated) allele frequency differences arising from ancestral source populations with distinct biogeographical origins.

### 2.5 Statistical analyses

All statistical analyses were performed using the R statistical language v3·6·1 [35]. T2D odds of prevalence were modeled using multivariable logistic regression computed using the ‘glm’ function in R. Age was standard normalized when included in logistic models. Two logistic regression models were used for analysis – Model 1: T2D ~ GA + SED + Age + Sex + GA*SED and Model 2: T2D ~ GA-SED + Age + Sex. It should be noted that Model 1 includes SED as a continuous variable and its interaction with GA, while Model 2 includes a categorical variable whose levels are given by the combination of GA categories and SED tercile categories, yielding a total of 9 categories with European Low SED as the reference. Odds ratios (ORs) and 95% confidence intervals were calculated for each term in the models by exponentiating the estimated coefficients. Forest plots were generated using the forestmodel R package [37]. The importance of predictors in the multivariable logistic regression was determined using dominance analysis [38] implemented in the R ‘dominanceanalysis’ v2·0·0 package. Dominance analysis estimates R^2^ values for all possible values of predictors and is used to measure the relative importance of predictors by running pairwise comparisons of all predictors in the model as they relate to the outcome variable. Linear regression equations and plots were generated using the R ‘ggplot’ v3·3·3 library. Slopes of linear regression models were compared by calculating a z statistic as described here [39].

### 2.6 Ethics approval

Ethics approval for the UK Biobank was obtained from the North West Multi-center Research Ethics Committee (MREC) for the United Kingdom, the Patient Information Advisory Group (PIAG) for England and Wales, and the Community Health Index Advisory Group (CHIAG) for Scotland (see https://www.ukbiobank.ac.uk/learn-more-about-uk-biobank/about-us/ethics).

### 2.7 Role of funding source

The funding sources did not have any role in study design, in writing of the report, or in the decision to submit the paper for publication.

## 3 Results

### 3.1 Type 2 diabetes ethnic disparities and socioeconomic deprivation

**Fig. 1:**
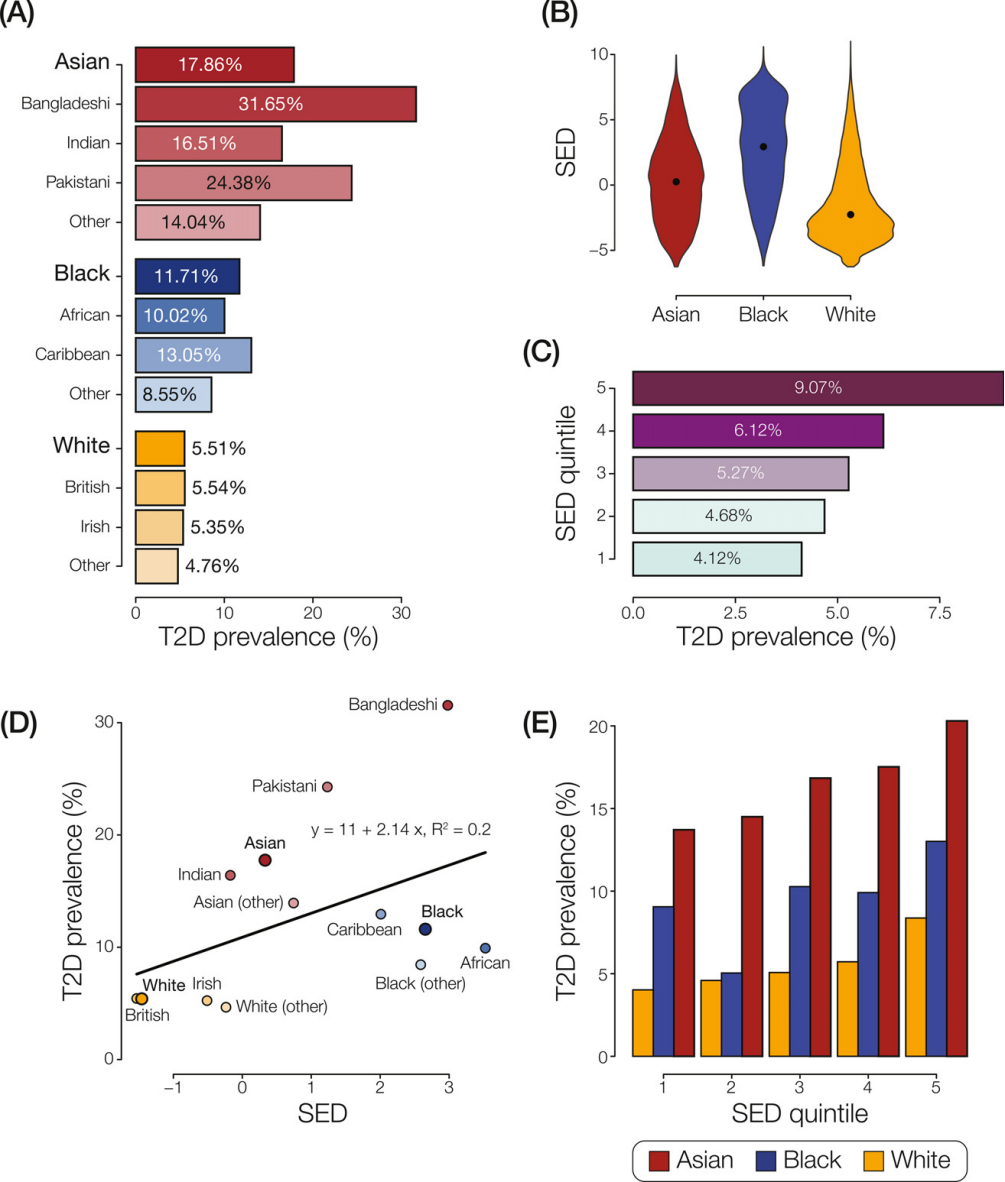
**T2 D ethnic disparities and SED.** (A) T2 D prevalence for ethnic groups and backgrounds. (B) SED distributions for ethnic groups. (C) T2 D for SED quintiles, 1-least deprivation to 5-highest deprivation. (D) Relationship between T2 D prevalence (y-axis) and mean SED (x-axis) for ethnic groups and backgrounds. (E) T2 D ethnic prevalence disparities across SED quintiles.

We generated type 2 diabetes (T2D) case/control cohorts from the UK Biobank using participants’ ICD-10 diagnosis codes, with the phecode scheme inclusion and exclusion criteria [33]. Our final analysis cohort had 27,748 T2D cases and 446,436 controls (Table 1). Participant case/control status was used to calculate T2D prevalence for the three largest ethnic groups in the UK – Asian, Black, and White – and for different levels of socioeconomic deprivation (SED). SED is measured using the Townsend index of deprivation, where lower values indicate less deprivation and higher values indicate more deprivation. It can be seen that T2D prevalence varies greatly among different ethnic groups and backgrounds in the UK (Fig. 1A). The Asian group shows the highest prevalence (17·86%) followed by the Black (11·71%) and White (5·51%) groups, respectively. The Asian group also shows the greatest variance of T2 D prevalence among constituent ethnic backgrounds. Within the Asian group, the Bangladeshi ethnic background shows the highest T2 D prevalence by far (31·65%), with the Indian (16·51%) and Other (14·04%) backgrounds showing prevalence values approximately half as high. Along with the ethnic disparity in the prevalence of T2 D, we also see a marked disparity in SED among the three groups under consideration (Fig. 1B). The Black group shows the highest level of median SED (2·93) followed by the Asian (0·25) and White (−2·27) groups, respectively. Consistent with what is known about the relationship between SED and T2 D, we also find that T2 D prevalence increases monotonically with an increase in social deprivation (Fig. 1C) [40,41].

**Table 1:** Characteristics of the T2 D analysis cohort.

Characteristic	Full cohort (*n* = 474,184)	Asian cohort (*n* = 9361)	Black cohort (*n* = 7541)	White cohort (*n* = 457,282)
Age – no. (Cohort share%)				
<45	47,697 (10·06)	1810 (19·34)	1611 (21·36)	44,276 (9·68)
45–54	133,102 (28·07)	3434 (36·68)	3359 (44·54)	126,309 (27·62)
55–64	201,760 (42·55)	2920 (31·19)	1807 (23·96)	197,033 (43·09)
>65	91,625 (19·32)	1197 (12·79)	764 (10·13)	89,664 (19·61)
Mean age – yr	56·62	53·32	51·90	56·77
Sex – no. (%)				
Female	257,015 (54·20)	4306 (46·00)	4309 (57·14)	248,400 (54·32)
Male	217,169 (45·80)	5055 (54·00)	3323 (42·86)	208,882 (45·68)
Median SED †	−2·19	0·25	2·93	−2·27
T2 D cases – no. (%)	27·748 (6·22)	1·672 (17·86)	883 (11·71)	25,193 (5·51)

† SED = Socioeconomic deprivation as measured with the Townsend index. Higher (positive) values of the index indicate high material deprivation, whereas lower (negative) values indicate relative affluence.

To further interrogate the relationship between SED and T2 D ethnic disparities, we compared the T2 D prevalence with the mean SED for each ethnic group and background (Fig. 1D). We see that a strong relationship does exist between group specific T2 D prevalence and SED, but the disparity is not completely explained by SED. Participants who identify as Black have higher average SED but a much lower T2 D prevalence compared to participants who identify as Asian, who have lower average SED compared to Black participants but far higher T2 D prevalence. Furthermore, on plotting T2 D prevalence per ethnic group for each SED quintile, we find that the ethnic disparities remain within each strata of SED, indicating that other factors also contribute to the T2 D ethnic disparities (Fig. 1E).

### 3.2 Genetic ancestry groups

Principal components analysis (PCA) of participants’ whole genome genotypes were used to generate discrete and coherent genetic ancestry (GA) groups. Overall, participants’ self-identified ethnicity co-varies with GA groups defined using PCA (Supplementary Figure 2). Nevertheless, there are numerous cases where participants’ self-identified ethnicity does not align with GA groups. Accordingly, we rely on GA group analysis to more precisely measure genetic differences that may be associated with T2 D ethnic disparities.

**Fig. 2:**
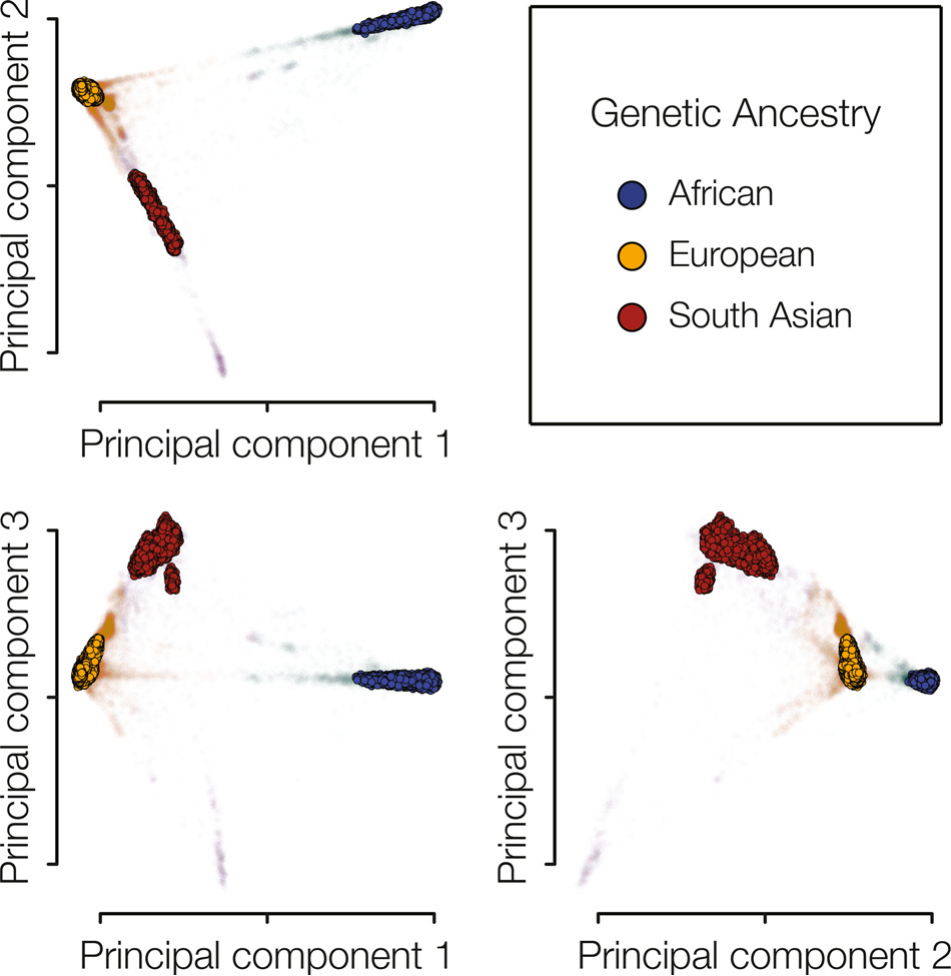
**GA groups.** Clustering of genetic PCA data was used to generate continuous and coherent GA groups: African (blue), European (orange), and South Asian (red). Participants that fall into coherent ancestry groups are prominently colored, and participants that fall into the continuous groups are shown as faded points. (For interpretation of the references to color in this figure legend, the reader is referred to the web version of this article.).

GA groups were delineated by performing density-based clustering of participant genetic PCA data, yielding three coherent groups: African (*n* = 5176), European (*n* = 448,446), and South Asian (*n* = 6969) (Fig. 2). The ancestral origins for these groups are based on the majority self-identification of member participants. These GA groups represent non-overlapping, discrete, and genetically diverse cohorts.

### 3.3 Genetic ancestry, socioeconomic deprivation, and type 2 diabetes

We modeled T2 D case/control status using GA and SED along with the covariates age and sex using multivariable logistic regression (Model 1; Supplementary Table 1). Model 1 includes SED as a continuous variable and its interaction with GA. This analysis shows that being part of the South Asian GA group compared to being in the European GA group had the highest impact on modifying T2 D risk (OR: 4·37, 95% CI: 4·10 – 4·66), followed by being in the African GA group (OR: 2·52, 95% CI: 2·23 – 2·85). As would be expected, age (OR: 1·78, 95% CI: 1·75 – 1·80), being male (OR: 1·86, 95% CI: 1·81 – 1·90), and SED (OR: 1·11, 95% CI: 1·10 – 1·11) are all significantly associated with T2 D risk. Dominance analysis shows that the most important predictors to explain T2 D status in this model are age, sex, GA group, and SED. However, we found the GA-SED interaction terms – South Asian-SED and African-SED – to be statistically significant (*p-*values of 0·001 and 0·016, respectively), suggesting that the impact of SED on T2 D varies among GA groups. The full model that includes the interaction term has a significantly higher log likelihood than a reduced model with no interaction term, further supporting the presence of GA-SED interactions (likelihood ratio ***χ***^2^ = 15·96 *P* = 3·4 × 10^−4^; Supplementary Table 2). Given the observed GA-SED interactions, it is not possible make any firm conclusions regarding the relative importance GA versus SED on T2 D outcomes.

**Fig. 3:**
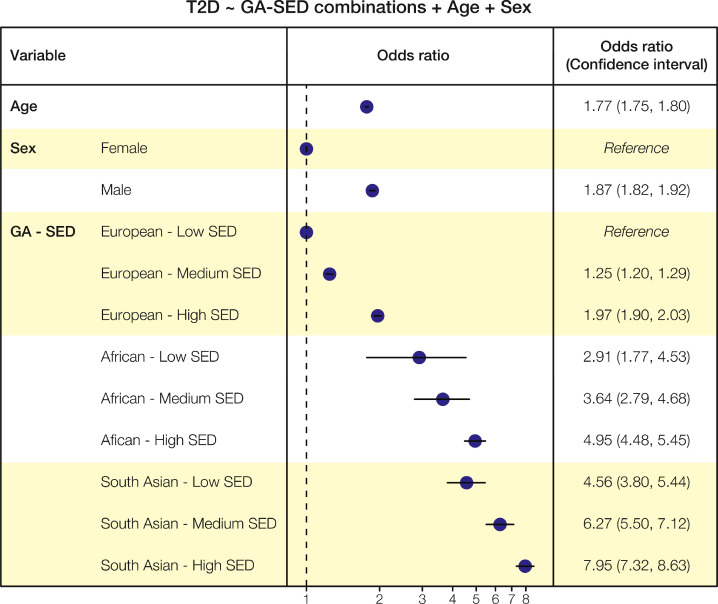
**T2 D multivariable logistic regression model with GA-SED tercile combinations (Model 2).** Model 2 includes terms for GA groups combined with low, medium, and high SED terciles, age, and sex. The forest plot shows odds ratios and 95% confidence intervals along with the statistical significance for each variable used to model T2 D status. Details of the estimated coefficients, their standard errors, and *p-*values are shown in Supplementary Table 3.

Next, we used another logistic regression model (Model 2; Supplementary Table 3), which includes a categorical variable whose levels are given by the combination of GA categories and SED tercile categories, yielding a total of 9 categories with European Low SED as the reference. As seen for Model 1, age, sex, GA and SED all show significant associations with T2 D status with Model 2 (Fig. 3). The relative impact of GA groups on T2 D status is the same: European has the lowest effect sizes, followed by African, and South Asian showing the highest effect sizes. For each GA group, increasing SED is consistently associated with greater effect sizes, thereby confirming the GA-SED interactions detected in Model 1.

**Fig. 4:**
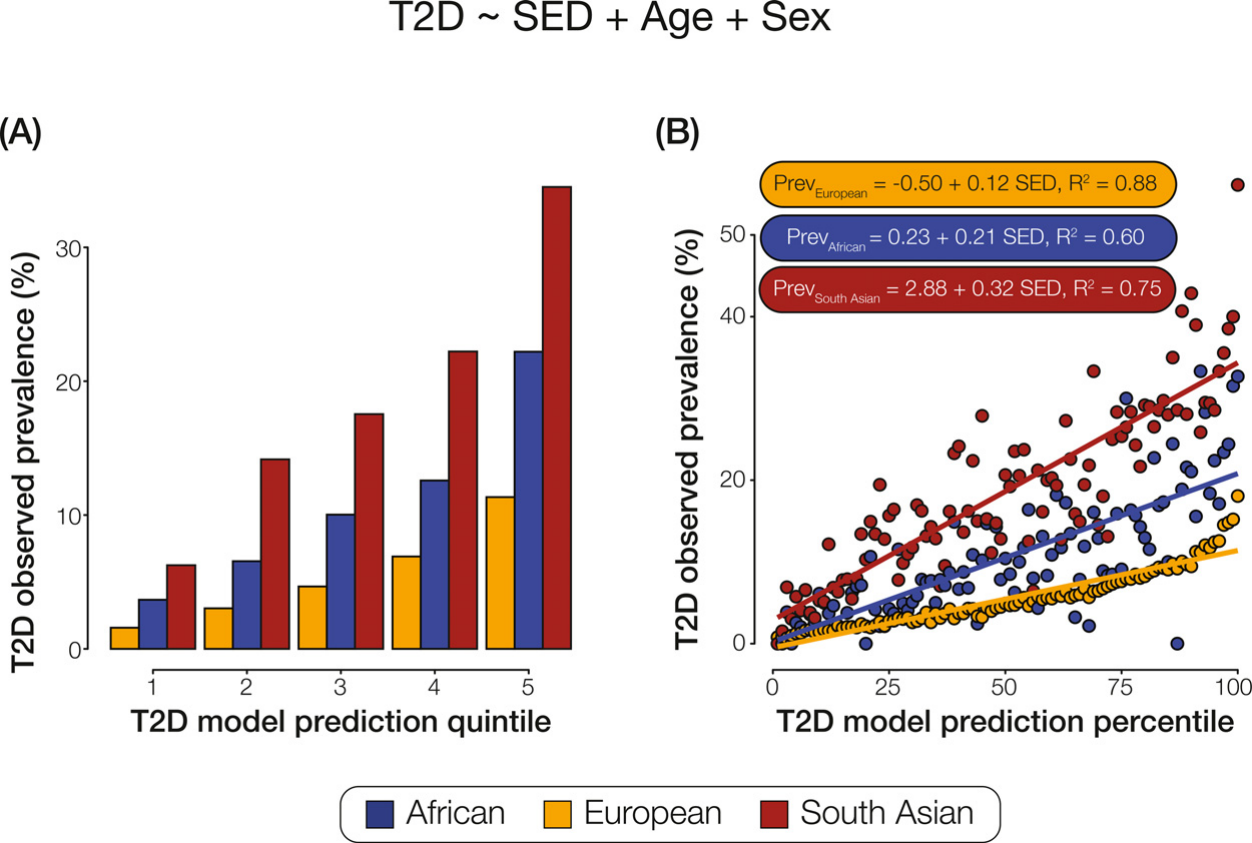
**Interaction between genetic ancestry and socioeconomic deprivation.** T2 D was predicted using a multivariable logistic regression model using SED, age, and sex as terms. T2 D prevalence per GA group partitioned by quintiles (A) and percentiles (B) of SED model predictions. Linear equations and model fits are shown for each ancestry group in panel B. Ancestry groups are color coded as shown.

To further characterize the impact of GA-SED interactions on T2 D status, we modeled T2 D case/control status using SED, along with the covariates age and sex, using multivariable logistic regression and then stratified the results by GA groups. For each individual GA group, the logistic model was used to calculate the probability of predicted T2 D per participant. T2 D observed prevalence values increase monotically for each GA group across T2 D model prediction quintiles, and the relative T2 D prevalence values for each GA group stay the same within each quintile (Fig. 3A). On regressing T2 D observed prevalence against T2 D model predictions per GA group and fitting a linear trend for each group separately, we found that the slopes for each GA group differed substantially (Fig. 3B). The magnitude of association between SED and T2 D for the South Asian group is ~2·5 times higher than the European group, and the African group association is ~1·5 times higher than the European group. Differences in the slopes are all statistically significant – confirming the interaction effects between GA and SED (African – European slope *p-*value = 7·28 × 10^−07^, African – South Asian slope *p-*value = 1·39 × 10^−05^, and European – South Asian slope *p-*value = 7·33 × 10^−25^). Furthermore, the intercept of this fitted line is higher for the South Asian group implying that even at the lowest possible SED level recorded in these data, the risk for T2 D is relatively high in this group.

## 4 Discussion

For this study on the UK Biobank, we confirmed previously observed T2 D ethnic disparities and found that SED is indeed a significant risk factor for T2 D. We report for the first time that T2 D is associated with a significant interactions between GA and SED. In particular, SED is a relatively greater risk factor for T2 D for individuals with South Asian and African ancestry, compared to those with European ancestry. This finding suggests that more ancestry-specific interventions need to be taken at the policy level to ameliorate health disparities, channeling resources to communities which are at highest risk.

We make a crucial distinction between GA and self-identified ethnicity in this study. As part of the UK Biobank enrollment survey, participants are asked to identify their ethnic group followed by their ethnic background (i.e., subgroup). For example, participants that identify with the Asian ethnic group are then prompted to choose from Bangladeshi, Indian, Pakistani, or Other Asian backgrounds. These self-identified ethnic group and background identities are social constructs based on shared heritage and culture, whereas GA reflects genetic differences among populations with distinct biogeographic origins. The approach of forming coherent clusters from genetic PCA data allowed us to generate discrete, non-overlapping GA groups, which can be used to help us disambiguate socioenvironmental factors from genetic factors that might contribute to T2 D ethnic disparities. It should be noted that the GA groups delineated here and the participant self-identified ethnic groups assess different constructs and are not entirely concordant (Supplementary Fig. 2). There are a number of cases where participants’ self-identified ethnicity does not coincide with their GA, but the majority of participants’ ethnic identities correspond to their GA. This reflects the fact that social determinants of ethnicity are strongly informed by notions of ancestral origins and may correlate with phenotypic characteristics.

SED is used here as a proxy for lifestyle factors and environmental exposures that might exacerbate or ameliorate risk for T2 D. The implications of a significant interaction between SED and GA groups can be attributed to a number of different factors. Lifestyle and exposures that co-vary with higher SED may have a disproportionately higher impact on T2 D risk in certain populations owing to their genetics, and/or higher SED may lead to different lifestyle and exposures among different populations. The latter possibility could include influences on SED-related experiences of structural oppression that differ among GA groups. In any case, targeted grou*p-*specific interventions that are informed by such differences can help to decrease T2 D health disparities.

There are several potential limitations to our observational study of T2 D health disparities. Some cultural attributes like diet and lifestyle factors might co-vary with GA, SED and self-reported ethnicity, especially for recent immigrants. Thus, the observed GA effects on T2 D could be attributed to unmeasured confounders. Co-variation between GA and environmental factors might change over time, with second and third generation immigrants becoming acculturated and changing their dietary habits. It has been shown that second generation Asians in England are more likely to be obese compared to the first generation of immigrants [42]. It is also known that the risk for T2 D in South Asians increases for a BMI >23 compared to a BMI of >25 in Europeans [43]. We did not account for generation of immigration in our GA analyses.

SED was measured here using the Townsend Index, which is a composite metric of four different variables, each of which may reflect different kinds of adverse exposures. This measure of SED may miss important indicators such as household income and education level. As this is an observational study, albeit with a large sample size, it is hard to completely disentangle the effects of different contributing factors on the observed health disparity. In addition, the UK Biobank recruited participants who are healthier, on average, compared to the general population and live in less socioeconomically deprived areas compared to non-participants (also referred to as a ‘healthy volunteer bias). Regardless, disease-exposure relationships in the UK Biobank are thought to be generalizable, irrespective of the healthy volunteer bias [44].

Finally, it should be noted that the PCA clustering approach used for GA inference yields groups that are largely concordant with continental ancestry. Accordingly, there is a substantial overlap between the GA groups analyzed here and participants’ ethnic self-identification (Supplementary Fig. 2). A more nuanced approach that includes quantitative GA estimates, i.e. percent ancestry contributions from ancestral source populations, could help to further disambiguate genetic from socioenvironmental effects on T2 D. Furthermore, the use of GA poses operational difficulties in targeting the impacted communities since this information is not readily available to policymakers and physicians. However, once a gene-by-environmental interaction is identified, as is the case here for the interaction between GA and SED, population-specific interventions and policies can be targeted at the closest corresponding ethnic groups where there exists a high concordance between GA and ethnic groups (Supplementary Fig. 2).

## Data sharing statement

This study was conducted using the UK Biobank resource under application number 65,206 granting access to LMR and IKJ to the corresponding UK Biobank biomarkers, and phenotype data. No additional data available. UK Biobank data is publicly available upon application on the UK biobank website (https://www.ukbiobank.ac.uk/register-apply/).

## Funding

This work was supported by the National Institutes of Health (NIH) Distinguished Scholars Program (DSP) to LMR and the Division of Intramural Research (DIR) of the National Institute on Minority Health and Health Disparities (NIMHD) at NIH. IKJ was supported by the IHRC-Georgia Tech Applied Bioinformatics Laboratory.

## CRediT authorship contribution statement

**Shashwat Deepali Nagar:** Conceptualization, Data curation, Formal analysis, Methodology, Visualization, Writing – review & editing. **Anna María Nápoles:** Funding acquisition, Methodology, Writing – review & editing. **I. King Jordan:** Conceptualization, Validation, Funding acquisition, Methodology, Project administration, Visualization, Writing – review & editing. **Leonardo Mariño-Ramírez:** Conceptualization, Validation, Funding acquisition, Methodology, Project administration, Writing – review & editing.

## Declaration of Competing Interest

The authors declare no competing interests.

## Appendix Supplementary materials

Image, application 1
